# Naturally Derived Cements Learned from the Wisdom of Ancestors: A Literature Review Based on the Experiences of Ancient China, India and Rome

**DOI:** 10.3390/ma16020603

**Published:** 2023-01-08

**Authors:** Zhan Su, Zhen Yan, Kazunori Nakashima, Chikara Takano, Satoru Kawasaki

**Affiliations:** 1 Division of Sustainable Resources Engineering, Graduate School of Engineering, Hokkaido University, Sapporo 060-8628, Japan; 2 Division of Sustainable Resources Engineering, Faculty of Engineering, Hokkaido University, Sapporo 060-8628, Japan

**Keywords:** naturally derived cement, Portland cement, enzyme-induced carbonate precipitation (EICP), microbially induced carbonate precipitation (MICP)

## Abstract

For over a thousand years, many ancient cements have remained durable despite long-term exposure to atmospheric or humid agents. This review paper summarizes technologies of worldwide ancient architectures which have shown remarkable durability that has preserved them over thousands of years of constant erosion. We aim to identify the influence of organic and inorganic additions in altering cement properties and take these lost and forgotten technologies to the production frontline. The types of additions were usually decided based on the local environment and purpose of the structure. The ancient Romans built magnificent structures by making hydraulic cement using volcanic ash. The ancient Chinese introduced sticky rice and other local materials to improve the properties of pure lime cement. A variety of organic and inorganic additions used in traditional lime cement not only changes its properties but also improves its durability for centuries. The benefits they bring to cement may also be useful in enzyme-induced carbonate precipitation (EICP) and microbially induced carbonate precipitation (MICP) fields. For instance, sticky rice has been confirmed to play a crucial role in regulating calcite crystal growth and providing interior hydrophobic conditions, which contribute to improving the strength and durability of EICP- and MICP-treated samples in a sustainable way.

## 1. Introduction

With the acceleration of urbanization, modern Portland cement is gradually becoming the most commonly used construction material due to its excellent properties [1,2]; however, modern Portland cement still has some noteworthy drawbacks. For example, the use of modern Portland cement in geotechnical reinforcement foundations increases the pH of the soil and surrounding groundwater. In addition, modern Portland cement is considered to have a fairly high carbon footprint. As the production of 1 ton of cement emits about 1 ton of CO_2_, the cement industry accounts for 5–8% of global CO_2_ emissions [3,4,5]. In 2021, cement consumption was expected to reach 4.4 billion tons, and its production was expected to generate 450 kg/m^3^ of CO_2_ emissions, representing 25% of total annual global manufacturing emissions [6,7,8] and 8% of anthropogenic CO_2_ emissions [9]. It is estimated that by 2050, global CO_2_ emissions from cement production will reach 2.34 billion tons [10]. In order to be more sustainable and reduce the environmental burden, a great deal of research has been conducted to develop new technologies to reduce the consumption of modern Portland cement in ground improvement practices.

With archaeological discoveries, there have been different types of calcareous and gypsum cements found that were widely used in buildings around the world as early as thousands of years ago [11]. Using specific natural additives, our early ancestors improved the performance of ancient cements to meet various needs. For example, the strength and durability of cement was more important when used to maintain living infrastructure construction or buildings that played an important role, such as city walls and harbors, and corresponding additives would be added [12]. Some of which gave these ancient cements extraordinary strength and durability, allowing ancient buildings to survive, even through thousands of years of environmental erosion.

Recently, similar to our ancestors’ practice of adding various additives to ancient cements, attempts have been made to use biopolymers in geotechnical engineering. Biopolymers are polymers produced from natural resources, including polysaccharides (e.g., cellulose), proteins (e.g., gelatin, casein and silk), and marine prokaryotes; biopolymers can also be produced by chemical synthesis of biologically derived monomers (e.g., polylactic acid) or microbial activities (e.g., xanthan gum or gellan gum) [3,13,14,15,16,17]. Biopolymers are environmentally friendly and have been widely used in food and medical applications [18,19]. Recent studies have shown how biopolymers can be used for soil consolidation [20,21,22,23,24,25], soil permeability control [26,27,28], erosion reduction [29,30,31,32,33,34], dust control [35,36,37,38] and even water treatment [39,40,41,42]. However, the durability of biopolymers is often questioned given the current limited yield of biopolymers and specifically regarding biopolymer-based soil treatments.

Meanwhile, the study of biocements has attracted many researchers [43,44,45,46]. This promising technology in specific geotechnical engineering could replace conventional methods for various situations, e.g., pre-construction soil improvement; slope and dam stabilization; stabilization of sandy soils; protection against wind and water erosion; waterproofing of ponds, canals, landfills and reservoirs; and chemical, radiological and biological soil immobilization. It could have a wide range of practical applications in the future [47]. There are two main types of biocement technology: MICP and EICP [48]. The mechanism of MICP is the use of the urea decomposition capacity of microorganisms to metabolize urea, producing ammonium and carbonate. The carbonate can then be combined with calcium ions to produce calcium carbonate precipitates (Equations (1)–(5)). Calcium carbonate precipitates can bind loose particles, strengthening and improving the strength and stiffness of the soil [49,50,51,52]. Similar to MICP, EICP also improves soil properties by inducing calcium carbonate precipitation. The difference is that EICP uses urease isolated from bacteria or plant solutions to carry out the reaction, and urease is a nickel-dependent metalloenzyme rather than a microorganism. However, biocement has some problems to overcome, such as the high cost of using EICP and MICP. In addition, one of the byproducts of the reaction, ammonium, is toxic to the natural environment and to humans. The combination of biopolymers and biocements may be a promising method to overcome the drawbacks from using biopolymers or biocements alone.

CO(NH_2_)_2_ + H_2_O → NH_3_ + NH_2_COOH (1)

NH_2_COOH + H_2_O → NH_3_ + H_2_CO_3_ (2)

2NH_3_ + 2H_2_O → 2NH_4_^+^ + 2OH^−^ (3)

2OH^−^ + H_2_CO_3_ → CO_3_^2−^ + 2H_2_O (4)

Ca^2+^ + CO_3_^2−^ → CaCO_3_ (5)

In this paper, we summarize and compare some ancient cement and modern principles of using biopolymer-reinforced foundations from different regions and try to link the high durability of ancient cements with the advantages of biopolymer-reinforced foundations and apply them to biocements. Thus, we try to solve the shortcomings of current biocements.

## 2. Types and Characteristics of Ancient Cements

### 2.1. The Wisdom of Ancient Rome

In the republican period of Rome, dated from the late 2nd century to mid-1st century B.C., the ancient Romans discovered how to create hydraulic cement, a kind of building material with exceptional performance [53,54]. Hydraulic cement was used as a durable building material. As an example, concrete structures in ports along the central Italian coast and in Mediterranean regions have maintained cohesion and integrity for 2000 years while partially or fully submerged in seawater. Consisting of lime and sandy volcanic ash, it is rich in chemically reactive aluminosilicates [55]. Pozzolanic reactions of volcanic ash with hydrated lime is thought to dominate the cementing fabric and durability of 2000-year-old Roman harbor concrete.

Ca^2+^ + 2OH^−^ + SiO_2_ → C-S-H (6)

Ca^2+^ + 2OH^−^ + Al_2_O_3_ → C-A-H (7)

Modern Portland cement is a crystalline-structured amorphous material made from clinker that is ground at 1450 °C, mixed with water and non-reactive aggregates (such as sand or gravel) and pumped into a mold in a fluid state. The concrete sets within a few hours and hardens within a few weeks as the Portland cement hydrates to form various compounds, mainly poorly crystalline calcium silicate hydrates (C-S-H), which bind to the sand and coarse aggregate (Equations (6) and (7)). Improper construction or maintenance results in uneven drying shrinkage of the hardened cement paste and concrete aggregates and microcracks in the concrete matrix, leading to a decrease in Young’s modulus and compressive strength. If the temperature is above 65 °C, uncontrolled hydration leads to the development of C-S-H, which absorbs sulfate and is subsequently expelled during cooling and aging to form deleterious ettringite, which causes the cement paste to swell and decouple from the aggregate, creating characteristic voids around these particles [56]. When extreme high temperature conditions are reached, Portland cement will begin to decompose [57]. This can significantly reduce the service life of Portland cement.

Compared with ordinary Portland cement, the amount of lime used in ancient Roman cement was only about 1/3 of that used in modern concrete; therefore, the amount of carbon dioxide emissions produced during the manufacturing of ancient Roman concrete was only about 1/3 of that of modern concrete. Meanwhile, the lifespan of Roman buildings is one to two orders of magnitude higher than that of modern buildings. Recent analytical techniques have shown that the remarkable durability of Roman hydraulic cements comes from the crystalline calcium aluminum silicate hydrate (C-A-S-H binder) in the cementitious matrix, which is produced by a reaction of seawater, lime, and volcanic ash [58,59,60,61]. One of the main components of Roman hydraulic cements, volcanic ash is a siliceous, aluminous material that has no cementing ability on its own, but can chemically react with water and calcium hydroxide to form compounds with cementing properties. When mixed with lime and rubble, they not only provide strength for other constructions, but when used for piers built in the sea, they solidify in the water and neither the waves nor the force of the water can dissolve them. Roman engineers soon realized the remarkable potential of this new material, as it was particularly suitable for building hydraulic installations, bridge footings and harbor structures. Roman engineers could build harbors wherever political, economic or military considerations existed, not limited to areas with favorable geographical features. The resulting changes in Roman architecture could only be described as “revolutionary”.

Al-tobermorite—a rare, layered calcium silicate hydrate mineral in ancient Roman cement—is composed of aluminosilicate chains bounded by interlayer regions and calcium oxide sheets [59]. Aluminum tobermorites are crystals with a plate-like shape, and these crystals are interlaced to strengthen the cementitious matrix, thus improving the resistance of concrete to brittle fracture. Researchers believe that as tobermorite grows throughout the concrete, it provides strength because of its long plate-like crystals that allow the concrete to flex without breaking when subjected to forces. Moreover, when the tobermorite (Al-tobermorite) forms between the aggregate and cement, it prevents further extension of microcracks, thus greatly enhancing durability [60]. 

Despite Roman cement having many disadvantages compared with modern Portland cement, such as its slower hardening time and the considerable time it takes for the seawater to strengthen the cement, as well as the final material being compressively weaker than Portland cement, its use has clear environmental advantages. The use of volcanic ash in Roman cement results in a reduced need for lime and correspondingly lower energy consumption and CO_2_ emissions than the Portland cement materials typically used today. Moreover, the longevity of Roman constructions is one to two orders of magnitude higher than that of modern constructions. Reinforced concrete (rebar) is used to construct massive structures in current construction, but its life expectancy is only several decades. Part of the reason for this is that when the surrounding concrete cures, oxidation takes place and the reinforcing bars rust over decades, causing sufficient expansion that causes cracks to form in the concrete. If the structure comes in contact with seawater, rebars could be corroded in less than 50 years, and reactions with calcium hydroxide would cause expansion within the concrete structure. Ancient Roman structures did not have steel reinforcement, but rather reinforced concrete on a structural scale. Roman cement was “self-healing” when it encountered seawater, meaning that when cracks appeared in the cement, the infiltrated seawater reacted with the phillipsite in the volcanic ash to form aluminium tobermorite crystals that filled the cracks and strengthened the whole [62,63,64]. Recently, some researchers have tried to apply the mechanism of Roman cement to improve modern cement. For instance, fly ash, a material which is produced by the combustion of coal, has been used for cement production. Fly ash has similar (pozzolanic) properties to the volcanic ash that Romans used to make their concrete, due to its broadly similar chemical composition; thus, it has greatly improved the strength and durability of concrete and has become a critical factor in the preservation of buildings [65].

### 2.2. The Wisdom of Ancient China 

Archaeological evidence suggests that lime-based cementitious materials were widely used to reinforce columns, make ground improvements and build roofs from the mid- to late Western Zhou Dynasty (1046–771 B.C.) [66]. By the time of the Northern and Southern Dynasties (386–589 A.D.) [67], glutinous lime cement was already a mature technology. Glutinous rice, also known as waxy rice, is a type of rice grown mainly in East and Southeast Asia. It is characterized by its white appearance, high straight-chain starch content, and sticky texture [68]. In addition to being a staple food in China, glutinous rice has been widely used in various other applications, including building construction. For example, many historical Chinese documents report the use of lime–sticky rice mortar for the construction of river dams, barns, food gates and tombs. The architectural work, *Tian Gong Kai Wu*, records in detail its composition, production methods and properties: “Cement was made by adding glutinous rice soup and actinomycetous sugar cane juice to a mixture of 1/3 lime and 2/3 loess and river sand, and mixing thoroughly. The buildings constructed with it are strong and durable, and this material is called Tabia.” This “Tabia” is the glutinous rice–lime cement mentioned above. The addition of natural organic compounds, such as glutinous rice soup, greatly improved the performance of cement building material. Due to its excellent properties and performance, such as high bond strength, good toughness, high water erosion resistance, and durability, sticky rice cement became the most established and widely used technology in ancient Chinese construction [69].

Modern evaluations show that the bonding properties of sticky rice cement are good enough to match modern cement. In addition to its high strength, sticky rice cement was characterized by its amazing durability [69]. It was used to build important architecture, such as city walls, palaces, stone bridges, dams, etc. After hundreds and even thousands of years, such architecture is still well-preserved. Further research have found that sticky rice pulp plays the role of a biological template, regulating and coordinating the carbonation of cement to generate nano-sized calcium carbonate crystals with a fine structure, and improves its toughness, impermeability, and compressive strength [70]. The reason why sticky rice can endure in cement for a long time is due to the anticorrosion effect of lime. The organic and inorganic compositions wrap and pad each other, similarly to the formation process of biomineralization products, such as bones, teeth, and shells. The partially reactive calcium hydroxide, wrapped in sticky rice pulp, inhibits the growth of bacteria and thus protects the glutinous rice from decay for a long time [71,72,73].

In addition to sticky rice soup, blood cement was also common in ancient China, and was mainly used for building painted floors [74]. According to relevant records, pig blood was used on the floor of the Xianyang Palace site during the Qin Dynasty (221–206 B.C.) [75], mixing lime and ginger stone into a dark red, smooth surface with a moisture-proof effect. It was found that animal blood played an important role in cement as it added air, reduced water, prevented freezing and thawing, resisted cracking and increased bond strength. The underlying mechanism is as follows: Firstly, the protein in the blood is expected to have a foaming ability. Thus, the tiny bubbles can improve the cement’s workability. Secondly, anions and hydrophilic groups in blood protein can generate electrostatic repulsion between cement particles and improve their dispersion. Thirdly, blood protein is decomposed in an alkaline environment, connects with calcium ions in cement, and enhances bond strength. Fourthly, the amino and carboxyl groups in blood protein provide waterproof ability.

Tung oil is another material that was widely used as a cement additive. This building material was mainly composed of boiled tung oil and lime mortar [76] because it had good water tightness, anti-codling effects and high bond strength. Thus, in ancient China, it was widely used in water well hooks, greasy seams in wooden boats, hole filling, housing grounds, and buildings with special requirements for waterproof ability and durability. According to recent research, the high performance of tung oil–lime cement was likely caused by the compact structure of this material. In this cement, calcium hydroxide reacts with tung oil and carbon dioxide, and produces calcium carboxylate and calcium carbonate, respectively. Thus, a lot of particles in mortar are bonded together through the coordination of calcium ions and crosslinking of tung oil, and a compact structure is formed [77]. This reaction between calcium hydroxide and tung oil was the most important factor causing tung oil–lime cement curing at an early stage. At this stage, the probability of calcium hydroxide being converted into calcium carbonate was very still very low, which is totally different from common pure lime cement; the early strength of tung oil–lime cement was given by the compact microstructure established by carboxylate. Even 10-year-old tung oil–lime mortar could not reach the relative degree of carbonization found in common lime mortar that had remained for 90 days [77]. The compact microstructure established by cement curing blocked carbon dioxide and water from entering the interior of cement. In addition, the hydrophobicity of tung oil also kept calcium hydroxide from water. As a result, tung oil–lime cement could keep alkalinity for a long time, further ensuring that the tung oil did not decompose and had long-lasting durability. 

### 2.3. The Wisdom of Ancient India 

The ancient Indians mainly used various plant and animal extracts to improve the strength and durability of ancient cements. For example, it was found from previous research literature that Indians added rennet, herbs, cactus extracts, lentils, castor oil, and many other natural animal or plant extracts to lime cement to build their temples, palaces and other important structures [78]. 

Several researchers have analyzed the effects of organic extracts and inorganic mineral additions used in lime cements in ancient India on mechanical properties [79]. The results showed that, in pure lime cement, for ease of construction, there are more water molecules between successive lime particles, which reduces bond strength and leads to early damage [80]. In addition, this may generate cracks in the lime cement and allow for their random propagation. Thus, under compressive conditions, pure lime fails earlier than cement with additives.

The addition of organic additives can change the properties of lime cements. For example, they increase the bond strength between particles in lime cements by enhancing adhesion, or by reducing the pore size. For example, the presence of proteins in the organic matter interacts with carbon dioxide to increase the hydrophobicity of the cement [81]. This interaction leads to the formation of calcium complexes, which increase compressive properties. In addition, fermented organic matter has air-entraining properties in the lime matrix. An organic matter additive can introduce millions of tiny air bubbles into the lime cement, improving the workability of the mix and thus reducing the necessary water/binder ratio, increasing its strength. In addition, when organics are added to lime cement, the entrained air promotes the carbonation process and this increased rate of carbonation increases the precipitation of carbonate crystals, leading to an increase in weight and mass, which ultimately increases the strength of the cement.

The addition of organic matter to the lime matrix could also affect the crystal core of calcium carbonate, changing the form of calcium carbonate or promoting the generation of other substances, thus changing the properties of the cement [80]. For example, when adding traditional herbal additives such as jaggery, the addition of organics in the lime matrix enhances the carbonation rate of lime and converts portlandite to form a new type of mineral, weddellite (calcium oxalate monohydrate). Formation of weddellite in the lime matrix can fill the gap between two lime particles and enhance the binding strength of mortar. Additionally, the calcium complexes formed during the interaction of proteins with the divalent calcium ions contribute to reduced water absorption, similar to synthetic polymers [81]. The proteinaceous material present in the lime mortar samples converts calcium oxide into calcium oxalate. These proteins can chemically react with clay particles by exchanging the inorganic cations of the clay with organic cations, resulting in a mechanism that uses the ability of amino acids (amides) to encourage clay flocculation. Therefore, organic material protects cement structures from environmental deterioration.

Overall, the carbohydrate, protein and fat compositions in different organic materials and their interaction with lime are important factors that affect compressive strength and increase bonding properties [82].

## 3. Future Prospects for New Ground Consolidation Technologies Learned from Ancient Cements

In general, additives used in cements throughout antiquity fall into two broad categories: organic and inorganic additives. In most cases, additives could contribute to strength improvements in strength and durability of the material by adjusting the crystal form, increasing viscosity, and reducing porosity.

In addition, it is worth noting that due to ancient productivity limitations, most additives were obtained directly from natural materials or from low-cost products and fertilizers from human life. This feature was a prerequisite to make an additive or cement widely available. Even royalty or religious leaders could not afford to use an additive for large-scale construction if the production costs were too high or the manufacturing process too complex to produce enough material.

Recently, similar to the ancestral practice of adding various additives to ancient cements, attempts have been made to start directly adding biopolymers for geotechnical engineering. Biopolymers are polymers produced from natural resources, including polysaccharides, proteins, chemically synthesized bio-derived monomers and microbial activity. Biopolymers are environmentally friendly and widely used in food and medical applications [26]. Table 1 summarizes common biopolymers used in geotechnical research and practice [83].

As one of the most prevalent natural biopolymers, starch is found in large quantities in the seeds, grains and roots of many different types of plants, including maize, rice, wheat, corn, potato and cassava. Depending on the source, this natural biopolymer has different characteristics and appearances [84]. Starch is used as thickeners and stabilizers [85], fortifying agents [86] and binders [87] in a variety of industries, including those in the food, textile, cosmetic, plastic, paper and pharmaceutical sectors. Starch has been utilized as a drilling fluid binder in the fields of geotechnical engineering and construction [88,89,90]. By cross-linking, it can increase the soil’s resistance to shear stress, and thus enhance the mechanical properties of the soil.

Xanthan gum is made up of two glucose, two mannose and one glucuronide that are mostly arranged in a helical pattern [91]. The viscosity of xanthan gum solutions increases linearly with increasing xanthan gum content and is highly stable over wide temperature, pH and electrolyte concentration ranges [23]. Due to its temperature stability, compatibility with food ingredients and pseudoplastic rheological properties, xanthan gum is widely used in the food industry [92]. Furthermore, xanthan gum is used in the petroleum industry as a gelling and suspending agent (flocculant) for viscosity control, as well as a thickening agent for drilling mud [93]. Recently, xanthan gum was found to be effective in increasing the shear strength and modulus of elasticity of soils, making them more suitable for use in foundation excavations and retaining walls. In these studies, the addition of small amounts of xanthan gum to soils was found to significantly increase their shear strength and modulus of elasticity. This makes xanthan gum a useful tool for improving the stability and bearing capacity of soils in geotechnical applications [94,95,96].

Guar gum is a neutral polysaccharide with random branching points of α-D-galactose units and a 1,4-linked β-D-mannopyranose backbone [97]. Foods frequently contain guar gum as a thickener, emulsifier or stabilizer. The ability of guar gum to hydrate quickly in cold water systems, producing highly viscous solutions even at low concentrations, is its most significant characteristic [18]. Guar gum solutions exhibit higher viscosity than xanthan gum solutions at the same biopolymer–water ratio [98]. Guar gum has been used to stabilize mine tailings in civil and geotechnical engineering by increasing their undrained shear strength by a factor of about 11 (2 to 22 kPa at 30% moisture content). It can also be used to prevent shallow cracking by stabilizing swollen soils on slopes and desert sands [99,100]. Additionally, it has been noted that guar gum slurry is utilized when building vertical barrier walls [101,102]. However, guar gum slurry can naturally decompose because of microorganisms or enzymes; as a result, durability becomes a crucial concern when using guar gum biopolymers in geotechnical engineering practice.

Eighty percent of the protein in cow’s milk is a phosphoprotein biopolymer called casein. Due to its hydrophobicity, casein biopolymers, which is a waste product of dairy and milk, have been used in a wide range of applications, including food, industrial coatings, adhesives, plastics and medical practices [103,104]. Casein has a higher wet strength when used in geotechnical and construction engineering practices because of its hydrophobicity.

Dextrose is a flexible biopolymer that can form coils with a high density and low level of permeability in aqueous media [105,106]. It is a homoglycan made up of glucose in linear chains connected by α-1,6-linkages. One of the first extracellular microbial polymers to be used in industry was dextran, which is frequently employed as a plasma extender [107]. Dextran was also utilized in tissue engineering [108,109,110]. The industrial isolation of plasma proteins, particularly albumin, immunoglobulins, proinsulin and other blood factors, is another significant application [111,112,113]. Dextran is also employed as an emulsifier in the food industry [114]. Dextran has been used as an additive in oil drilling mud [115,116] and as a soil stabilizer; it is an effective soil aggregate in civil and construction engineering. According to some reports, dextran increases the proportion of aggregates (>75 m) and changes the size distribution of microaggregates [117].

Chitin, found in insect, squid and crustacean shells, is converted into the linear polysaccharide chitosan by deacetylation. Human cells can tolerate chitosan, which has no adverse effects on the immune system. In order to thicken, stabilize and manufacture food and biological materials, chitosan is widely used. In earthen construction, chitosan has been introduced as a workable and sustainable additive [42]. Chitosan’s cationic charge interacts electrostatically with the negative charges of clay particles to produce condensates in clay suspensions [15,118,119] and faceted packing of clay deposits [120]. Chitosan wraps around sand particle surfaces to improve waste removal through pore plugging, which significantly lowers the hydraulic conductivity of the soil for soil remediation [26,121].

Agar gum is frequently used as a gel thickener and food stabilizer because it is made of linearly linked galactose molecules [106]. Agar gums can also be used for drug therapy [122,123] and as culture media for genetic and microbiological research [124,125]. Agar gum is generally derived from various species of Rhodophyta (red algae), and it has recently been used as a low environmental load additive to increase soil strength. By gelating, agar gum can produce significant quantities of soil–biopolymer aggregates. Agar gum also has a longer molecular structure, which enables it to coat and coagulate soil particles, thereby enhancing soil strength.

**Table 1:** Common biopolymers used in geotechnical engineering.

Biopolymers	Composition	Cost [$/kg]	Source	Reference
Starch	C_27_H_48_O_20_	1–5	Seeds, grains and roots of plants	[84,86,87,88,89,90]
Xanthan Gum	C_35_H_49_O_29_	2–5	Xanthomonas campestris	[23,91,92,93,94,95,96]
Guar Gum	C_10_H_14_N_5_Na_2_O_12_P_3_	1–30	Cyamopsis tetragonoloba	[18,92,93,97,98,99,100,101,102]
Casein	C_81_H_125_ N_22_O_39_P	5–50	Milk	[103,104]
Dextran	C_18_H_32_O_16_	15–60	Lactic acid bacteria	[105,106,107,108,109,110,111,112,113,114,115,116,117]
Chitosan	C_18_H_35_N_3_O_13_	10–100	Insects, squid bones, and crustacean shells	[15,39,42,118,119,120,121]
Agar Gum	C_14_H_24_O_9_	10–100	Rhodophyta	[22,106,122,123,124,125]

**Figure 1:**
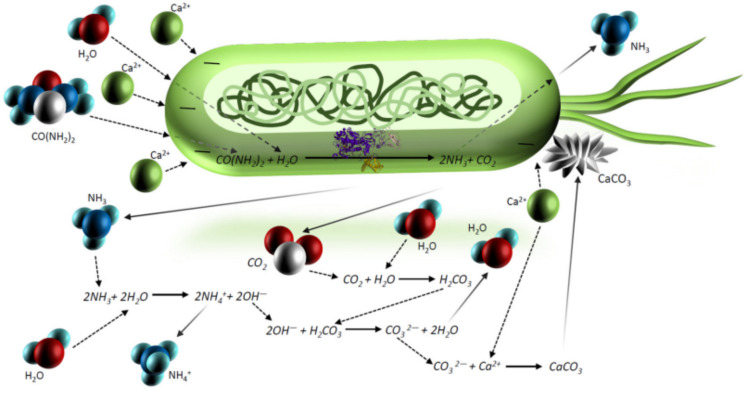
A representation of calcite precipitation due to the MICP method [48].

**Figure 2:**
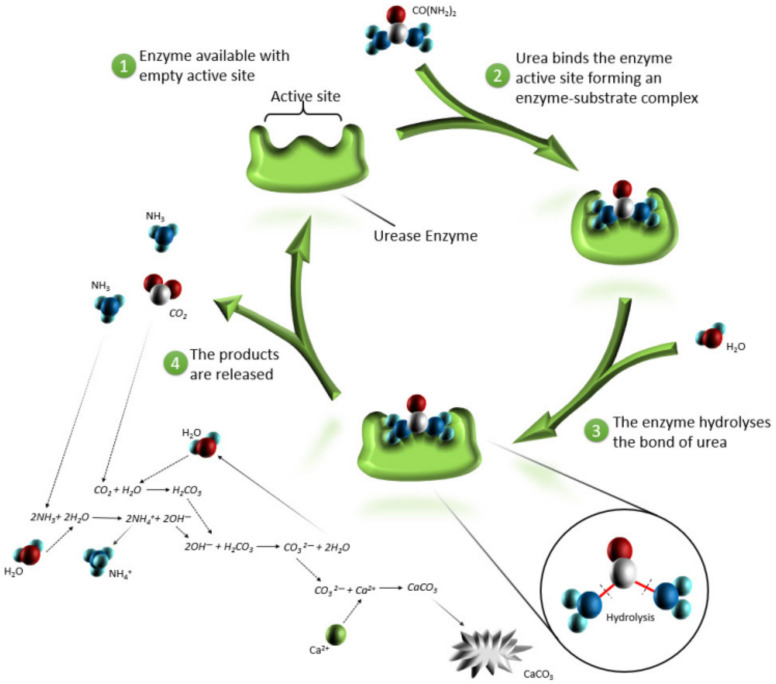
A representation of calcite precipitation due to the EICP method [48].

In recent years, the rise in sustainable development has promoted the development of biocements in the geotechnical field. Biocement technology is at the intersection of the natural environment and architectural disciplines, which have a significant impact on the economy, society and environment and broad prospects. As mentioned above, there are two types of biocement technologies: EICP and MICP. The mechanisms of these two technologies is to induce calcium carbonate precipitation in the soil matrix using microorganisms (Figure 1) or urease (Figure 2), respectively. The connection of calcium carbonate particles and matrix particles can improve soil properties.

From ancient cements and modern biopolymers comes the possibility of using some low-cost materials from our natural resources and productive life as additives to improve the properties of biocements. Compared with traditional technologies, biocement has many advantages, such as less carbon dioxide emissions and low-pressure injection work [126,127]. It is considered a promising technology and has been actively studied over the past two decades. However, both EICP and MICP technologies have some problems. For example, the costs are relatively high, and it is difficult to accurately control the intensity and uniformity [128].

Therefore, some researchers have tried to use some low-cost production and domestic wastes as a calcium source, ammonium source, or additives for reactions in experiments, hoping to control costs and strengthen performance of improved soil. For example, solid leather waste has been used to promote the carbonate precipitation process [129]. Lime solution was used as a substitute for a calcium source, and the leather hydrolysate powder obtained from thermal hydrolysis of leather waste residues was used to produce urease for the EICP reaction. As a result, production costs were reduced by about 51.4%. In addition, the use of meat waste reduced the total amount of solid waste produced during leather processing by approximately 21.77%. The consumption of suspended matter in lime solution can also reduce the pollution load by 31.95%.Thus, the utilization of low-cost leather industry waste for EICP could reduce costs and protect the environment.

Additionally, conducting MICP experiments with low-grade products instead of the high-grade chemicals that are often used in the laboratory is another method to control costs [130]. Compared with using pure, lab-grade chemicals as raw materials, a group using low-grade materials showed higher UCS strength. According to the SEM images, the combination of soil particles and calcium carbonate precipitations were widely observed in these samples. The unusual formation of a dense matrix is due to the presence of other polymer substances (PS) in low-grade chemicals. When calcite precipitation occurs, the precipitate encapsulates the PS and fills the void spaces, effectively providing necessary matrix support. By replacing pure chemicals with low-grade chemicals, a significant improvement in the UCS of soil was obtained, together with a 96% reduction in treatment costs.

Some researchers used jute fiber as an additive and found that jute fiber had significant effects on microbial performance, calcium carbonate precipitation patterns and sand solidification [131]. Fluorescence microscopy showed that the addition of jute fiber obviously improved the viability of microorganisms. The amount and length of jute fiber effectively improved the bacterial properties and mechanical properties (UCS and ductility) of sand, resulting in an increase in UCS with an increase in fiber content. However, when the amount of added fiber exceeded a certain point (3% and 15 mm), entanglement between fibers easily occurred, which hindered the entry of bacteria and reduced their living space, thus decreasing the formation of calcium carbonate and eventually reducing UCS. SEM analysis showed that the added jute fibers coupled well with calcium carbonate crystals and formed a reliable bridge within the soil matrix, limiting the development of failure surfaces inside the specimen and improving the mechanical properties of the specimen.

Overall, the use of low-cost materials from natural resources and productive life as additives for improving biocement performance should be further studied and discussed. Additives used in ancient cements, such as glutinous sticky rice and volcanic ash, were shown to improve some properties of calcium carbonate crystallization at the micro level and thus improve the performance of lime cement. In addition, these additives have many advantages, such as low cost, being easy to obtain from the natural environment, being harmless to the environment and so on, which meet the requirements for sustainable development. Therefore, this paper summarizes some additives used in ancient civilizations and makes the following conclusions: As mentioned above, there have been many experimental studies on additives in microbial cement using low-cost natural materials or wastes, but most of them are derived from modern chemical materials or products. Research on whether low-cost additives in ancient lime cement can be applied to the field of biocement is rare. Moreover, given that most buildings built by ancient lime cement have survived hundreds or thousands of years under natural erosion, the use of the same kind of additive may contribute to remarkable anti-corrosion properties, such as water erosion resistance. We recommend conducting a hybrid study that combines ancient cement technology with current biocement technology to overcome the high costs, low strength and durability problems of biocement usage.

## 4. Conclusions

The current consensus in geotechnical engineering is to adopt low-cost, environmentally friendly technologies for sustainable development. This paper summarizes the types and specific additives of ancient cement and draws several conclusions.

Lime cement has a long history. Additives can significantly alter the properties of lime cement and have allowed ancient architectures to be preserved for hundreds of thousands of years. The ancient Romans improved the strength and watertightness of lime cement by adding volcanic ash. It has been shown that volcanic ash can form a new crystal structure of tobermorite in cement, which presents a plate-like structure and can increase the toughness of concrete and improve the structure’s mechanical properties. The ancient Chinese made their cement mainly by adding glutinous sticky rice and some other animal and plant products, of which glutinous rice cement was the most brilliant. On a microscopic level, glutinous rice can regulate calcium carbonate crystals to form tiny, dense structures and wrap around them to fill the gaps between them, thereby reducing porosity and increasing strength. In addition, the alkaline environment in cement can effectively help the starch from breaking down over a long period of time, thus improving the life of the entire structure. The ancient Indians used plant and animal products to make additives. Plant juice promotes calcium oxalate production, whereas animal products contain ingredients such as protein and animal glue to fill gaps and improve performance.

Overall, the additives used in ancient cements improved the performance of lime cement. Most of these additives are low-cost, environmentally friendly, easy to obtain and so on, meeting the needs of the geotechnical engineering field, today and for the future. In addition, as one of the fields of geotechnical engineering, biocement technology has many advantages, and its future development is promising. From a sustainable engineering perspective, this soil stabilization technology is not only a practical resource/waste management approach, but also contributes to the creation of countless jobs. Therefore, this paper summarizes some additives used in ancient civilizations, making valuable suggestions for future biocement technology in the selection and research of additives.

## Data Availability

No new data were created or analyzed in this study. Data sharing is not applicable to this article.
